# Full-Genome Sequence of the First G8P[14] Rotavirus Strain Detected in the United States

**DOI:** 10.1128/genomeA.00677-15

**Published:** 2015-06-18

**Authors:** Slavica Mijatovic-Rustempasic, Sunando Roy, Michele Sturgeon, Kunchala Rungsrisuriyachai, Erik Reisdorf, Margaret M. Cortese, Michael D. Bowen

**Affiliations:** a Division of Viral Diseases, National Center for Immunization and Respiratory Diseases, Centers for Disease Control and Prevention, Atlanta, Georgia, USA; b Wisconsin State Laboratory of Hygiene, Madison, Wisconsin, USA

## Abstract

This is a report of the complete genomic sequence of a rare rotavirus group A G8-P[14]-I2-R3-C2-M2-A3-N2-T6-E2-H3 strain detected in a stool sample from a 57-year-old subject.

## GENOME ANNOUNCEMENT

Group A rotaviruses (RVA) are a major etiologic agent of gastroenteritis in infants and young children worldwide (1). Among adults, RVA infections have been associated with nonseasonal outbreaks, with a wide spectrum of disease severity (2). The RVA genomic classification nomenclature is based on all 11 segments of double-stranded RNA (dsRNA) encoding the VP7, VP4, VP6, VP1-3, and NSP1-5/6 proteins and uses the notation Gx-P[x]-Ix-Rx-Cx-Mx-Ax-Nx-Tx-Ex-Hx, respectively, with “x” indicating the numbers of the corresponding genotypes (3). For a majority of human RVA strains, variation in the backbone genes can be differentiated by three genotype constellations, the Wa-like genogroup 1 (I1-R1-C1-M1-A1-N1-T1-E1-H1), the DS-1-like genogroup 2 (I2-R2-C2-M2-A2-N2-T2-E2-H2), and the AU-1-like genogroup 3 (I3-R3-C3-M3-A3-N3-T3-E3-H3) that are believed to have originated from porcine, bovine, and feline RVAs, respectively (3).

Here, we report the full-genome sequence of RVA strain RVA/Human-wt/USA/2012841174/2012/G8P[14] (2012841174), detected in 2012 in a stool sample from a 57-year-old subject from Wisconsin. The methods used for determining the full-length genome sequence and assignment of genotypes were described previously (4). The sizes of full-length segments 1 to 11 were 3,302, 2,684, 2,591, 2,362, 1,578, 1,356, 1,075, 1,059, 1,062, 751, and 667 bp, respectively, and the open reading frame lengths for these segments were 3,267, 2,643, 2,508, 2,321, 1,476, 1,194, 933, 954, 981, 528, and 597 bp, respectively.

The strain 2012841174 genome constellation is G8-P[14]-I2-R3-C2-M2-A3-N2-T6-E2-H3 and differs from other previously reported G8P[14] strains because the VP1 (R3) and NSP1 (A3) genes are from genogroup 3. Phylogenetic analysis of individual genes revealed a combination of genes of animal and human origin. The VP2, VP3, VP6, NSP1, NSP3, and NSP4 genes share a high degree of nucleotide homology with bovine strains (95.4 to 97.5%), while the VP1, NSP2, and NSP5 genes are closely related to simian strains, with nucleotide similarities ranging from 97.5 to 98.8%. The VP7 and VP4 genes share 87.4% and 96.2% nucleotide identity with human strains WAG8.2 and A64, respectively.

Recent phylogenetic analysis revealed seven distinct lineages among P[14] strains (5), and the P[14] gene of strain 2012841174 falls into lineage 1. G8 RVA strains in combination with P[14] have been known to infect children and cattle in several countries (6–9). There is limited genetic information regarding these strains, since only 4 full-genome sequences for G8P[14] strains have been reported to date. This is the first G8P[14] strain identified in the United States.

### Nucleotide sequence accession numbers.

The strain 2012841174 gene sequences have been deposited in GenBank under the accession numbers KJ411432 to KJ411442.
